# Natural killer cell subset count and antigen‐stimulated activation in response to exhaustive running following adaptation to a ketogenic diet

**DOI:** 10.1113/EP090729

**Published:** 2023-02-26

**Authors:** David M. Shaw, Lauren Keaney, Ed Maunder, Deborah K. Dulson

**Affiliations:** ^1^ School of Sport, Exercise and Nutrition Massey University Auckland New Zealand; ^2^ Sports Performance Research Institute New Zealand (SPRINZ) Auckland University of Technology Auckland New Zealand; ^3^ School of Biomedical, Nutritional and Sport Sciences, Faculty of Medical Sciences Newcastle University Newcastle Upon Tyne UK

**Keywords:** CD56, CD69, endurance, exercise, flow cytometry, ketosis, lymphocytes

## Abstract

We investigated the effect of a 31‐day ketogenic diet (KD) compared with a habitual, carbohydrate (CHO)‐based diet on total circulating natural killer (NK) CD3^−^CD56^+^, dim and bright subset count, and antigen‐stimulated CD3^−^CD56^+^ cell activation (CD69^+^) in response to exhaustive running. In a randomised, repeated‐measures, cross‐over study, eight trained, male endurance athletes ingested a 31‐day low‐CHO KD or their habitual diet (HD). On day 31, participants ran to exhaustion at 70% ${\dot{V}}_{O_{2}\max}$ (∼3.5–4 h, ∼45–50 km). A low‐CHO (<10 g) meal was ingested prior to the KD trial, with fat ingested during exercise. A high‐CHO (2 g kg^−1^) meal was ingested prior to the HD trial, with CHO (∼55 g h^−1^) ingested during exercise. Venous blood samples were collected at pre‐exercise, post‐exercise and 1 h post‐exercise. The KD amplified the classical exercise‐induced biphasic CD3^−^CD56^+^ cell response by increasing the post‐exercise counts (*P* = 0.0004), which appeared to be underpinned by the cytotoxic CD3^−^CD56^dim^ subset (main effect of time point, *P* < 0.0001). The KD had no effect on NK cells’ expression of CD69 or their geometric mean fluorescence intensity of CD69 expression, either for unstimulated or for antigen‐stimulated NK cells (all *P* > 0.05). In conclusion, adaptation to a KD may alter the number of circulating NK cells but not their ability to activate to an antigenic challenge.

## Abstract (highlights)

- **What is the central question of this study?**
  Does a ketogenic diet (KD) modulate circulating counts of natural killer (NK) cells, including CD56^bright^ and CD56^dim^ subsets, and their ability to activate (CD69 expression) following in vitro antigen stimulation in response to exhaustive moderate‐intensity exercise?
- **What is the main finding and its importance?**
  The KD amplified the biphasic exercise‐induced NK cell response due to a greater mobilisation of the cytotoxic CD56^dim^ subset but did not alter NK cell CD69 expression. The KD appears to modulate exercise‐induced circulating NK cell mobilisation and egress, but not antigen‐stimulated circulating NK cell activation.

## 1 INTRODUCTION

Endurance athletes are thought to be more susceptible to illness due to engaging in prolonged, exhaustive exercise that transiently perturbs immune components suggestive of immunosuppression (Walsh et al., 2011). This tends to be exacerbated by low‐carbohydrate (CHO) availability due to a greater increase in the exercise‐induced glucocorticoid and catecholamine stress response (Bermon et al., 2017). Maintaining high‐CHO availability during exercise is, therefore, promoted to augment immunocompetency (Bermon et al., 2017). Very low‐CHO ketogenic diets (KDs) conflict with this paradigm and only two studies have assessed their impact on immune responses to exercise (McKay et al., 2018; Shaw et al., 2021), while their effect on illness risk is largely unknown. Recently, we demonstrated that adaptation to a 31‐day KD does not appear to suppress in vitro T‐cell‐related cytokine responses to multi‐antigen stimulation at rest and in response to exhaustive running (∼3.5‐4 h) in trained, male endurance athletes (Shaw et al., 2021); however, these acquired immune responses do not reflect immune cells of the innate immune system, which act as our first line of defence against pathogens.

Natural killer (NK) cells of the innate immune system can respond differently to T‐cells following identical dietary exercise interventions (Fletcher & Bishop, 2012) and are sensitive to changes in CHO‐availability (Henson et al., 1999). NK cells are large granular lymphocytes that play a critical role in the first line of defence against pathogens and neoplastic cells (Caligiuri, 2008). They are identified by the cell surface marker CD3^−^CD56^+^ and can be divided into subsets (Caligiuri, 2008). Generally, CD56^bright^ NK cells, which comprise ∼10% of total circulating NK cells, appear to be important for coordinating ‘cross‐talk’ between the acquired and innate arms of the immune system due to their capacity to produce cytokines, whereas CD56^dim^ NK cells are more cytotoxic and can lyse cells without antibody recognition (Caligiuri, 2008). CD56^dim^ NK cell mobilisation also appears sensitive to adrenergic stimulation (Dimitrov et al., 2010), suggesting the greater adrenergic response from a KD could alter exercise‐induced NK cell trafficking patterns from secondary lymphoid to peripheral tissues via the blood and therefore amplifying the biphasic NK response.

Further, upon activation, NK cells express CD69 (i.e., early activation marker) (Borrego et al., 1999; Werfel et al., 1997). CD69 expression is important for NK cell function, including roles in cell proliferation, cytolytic activity and cytokine secretion (Borrego et al., 1999; Werfel et al., 1997). Only ∼10% of unstimulated peripheral blood NK cells express CD69, which increases several‐fold upon antigen stimulation (Werfel et al., 1997). However, the relationship between NK cell CD69 expression, exercise and CHO‐availability is not well‐understood. Despite NK cell function typically being impaired by increased cortisol concentrations (Muscari et al., 2022), the relationship between corticosteroids and NK cell CD69 expression is less clear, with little work currently conducted in this area.

Further investigation into the effect of a KD on immune function is warranted, particularly in populations engaging in exhaustive exercise lasting several hours, who may be at an increased risk of illness. Therefore, we continued our investigation into the effects of a KD on immunity through examining circulating NK cells in response to exhaustive running in trained male endurance athletes. We hypothesised that a KD would increase total NK cells and the CD56^dim^ NK cell subset following exercise but suppress CD3^−^CD56^+^ NK cell activation (expression of CD69) following in vitro multi‐antigen stimulation during recovery. This study is part of a series assessing the effects of a KD on performance and health (Maunder et al., 2021; Shaw et al., 2019, 2021).

## 2 METHODS

### 2.1 Ethics approval

The study was performed according to the ethical standards established by the *Declaration of Helsinki* 2013 and was approved by the Auckland University of Technology Ethics Committee (17/410, approved 11 December 2017) (Auckland, New Zealand). The study was not registered in a database. All participants signed an informed consent form prior to participation.

### 2.2 Participants

Participants were required to have been: (1) male; (2) habitually consuming a mixed diet for >12 months; (3) weight stable for >1 month; (4) running >50 km per week; and (5) completed a marathon in <3.5 h within the previous 6 months. Only males were included to avoid the potential effect of the menstrual cycle and various types of contraception on immune and physiological indices. Participants were excluded if they: (1) reported a history of fat‐ or keto‐adaptation (i.e., intentionally reduced dietary CHO intake <150 g per day for several weeks in order to amplify exercising fat oxidation); (2) previously ingested exogenous ketone supplements; (3) were currently or recently injured; (4) experienced moderate‐to‐severe gastrointestinal symptoms or illness within the previous 4 weeks; (5) had a history of irritable bowel syndrome; (6) habitually smoked; or (7) had been ingesting dietary supplements or medications known to affect immune function within the previous 2 weeks, with the exception of caffeine, protein and CHO supplements. All characteristics were acquired by self‐report. Eight eligible healthy, trained, male endurance athletes (two marathoners, four ultra‐marathoners and two long‐distance triathletes) participated in the study. Participants were fully informed of the study's rationale and possible risks of the experimental procedures before providing their written consent.

### 2.3 Experimental overview

A detailed overview of this study's design, experimental trials, dietary intervention and monitoring, and training has been published previously (Shaw et al., 2019). The study was conducted in New Zealand during autumn and winter following the participants’ competitive running season when participants’ training load was low‐to‐moderate. In brief, participants underwent two open‐label 31‐day experimental conditions (i.e., KD or habitual diet (HD)), separated by a 14‐ to 21‐day washout period, in a randomised (www.randomizer.org), counterbalanced, crossover design. Whilst pre‐ and post‐diet run‐to‐exhaustion trials were conducted, only samples from the post‐diet trials were used to analyse NK cell responses. An incremental exercise test to exhaustion was performed 2 days prior to each trial to examine changes in maximal oxygen uptake (${\dot{V}}_{O_{2}\max}$). All interventions, trials and analyses were conducted by the same researcher. A registered dietitian prescribed the KD (≤50 g per day CHO, 15–20% energy intake (EI) from protein and 75–80% EI from fat) and educated and monitored each participant for compliance. Nutrient intake for each dietary condition was determined using in an image‐assisted, weighed diet record reported remotely in real‐time, with frequent measurement of blood and urinary ketones. Participants were requested to refrain from alcohol and dietary supplement use for the duration of the study. Participants also designed their own 28‐day training schedule and were asked to replicate this during each diet.

To assess the effect of diet on the immune response to exercise, participants were requested to rest following their incremental exercise test (∼48 h) and present to the laboratory between 06.00 and 07.00 h having abstained from caffeine for the previous 24 h. In the KD trial, participants continued ingesting a KD following the incremental exercise test and ingested a prescribed low‐CHO (<10 g CHO), high‐fat breakfast 90 min prior to the trial. In the HD trial, participants were requested to ingest a high‐CHO diet following the incremental exercise test and ingested a prescribed iso‐energetic breakfast containing 2 g kg^−1^ of CHO 90 min prior to the trial. Upon arrival to the laboratory, an indwelling intravenous Teflon catheter (18G, Terumo, Tokyo, Japan) was inserted into the antecubital vein for serial venous blood sampling and body mass was measured (shorts only). Participants then commenced running at the speed estimated to elicit 70% ${\dot{V}}_{O_{2}\max}$ until volitional exhaustion. In the KD trial, participants received a combination of artificially sweetened fluids (electrolyte drink and cordial), water and coconut oil (Blue Coconut Oil, Blue Coconut, Christchurch, New Zealand); the fluid and energy ingested during each trial were matched. In the HD trial, participants were prescribed 4 ml kg^−1^ of a 7.2% CHO‐electrolyte drink (Replace, Horleys, Masterton, New Zealand) every 20 min, which was adjusted based on each participant's tolerance (∼55 g CHO h^−1^). Immediately following exercise cessation and sample collection, participants had their body mass weighed, were provided with 5 ml kg^−1^ of water and rested for 1 h.

### 2.4 Blood sampling and analysis

Venous blood samples were collected at pre‐exercise, post‐exercise and 1h post‐exercise into an a 6 ml sodium heparin and a 6 ml K_2_EDTA Vacutainer (BD, Franklin Lakes, NJ, USA) with the participants seated in an upright position. Due to the rapid exercise‐induced lymphocyte kinetics (Rooney et al., 2018), all blood samples were collected within 1 min of the specified time points. The cannula was flushed with 3–4 ml of saline every 30 min to maintain patency. Each serum vacutainer was left to clot for 30 min prior to centrifugation at 1500 *g* for 10 min at 4°C. After this, samples were separated into two 1.5 ml aliquots to be stored at –80°C prior to the analysis of glucose and cortisol concentration (Cobas Modular P800 Analyser, Roche Diagnostics, Auckland, New Zealand). Capillary blood d‐β‐hydroxybutyric acid (d‐βHB; Freestyle Optium Neo, Abbott Diabetes Care, Doncaster, Victoria, Australia) concentration was measured at pre‐exercise, post‐exercise and 1‐h post‐exercise from a fingertip blood sample using standardised techniques. Glucose, d‐βHB and cortisol concentrations have been previously published (Shaw et al., 2021).

### 2.5 Haematological analysis

K_2_EDTA treated whole‐blood was used to determine red blood cell, haematocrit, haemoglobin and total lymphocyte concentration (Sysmex XT‐2000i Automated Haematology Analyzer, Sysmex Corp., Auckland, New Zealand). All cell concentrations were adjusted for plasma volume changes from the pre‐exercise blood sample using the formula: [Hb_pre_ × (1 – Hct_post_)]/[Hb_post_ × (1 – Hct_pre_)]) – 1, where Hb is haemoglobin and Hct is haematocrit (Dill & Costill, 1974).

### 2.6 Multi‐antigen‐stimulated whole‐blood cultures

Blood samples from the sodium heparin vacutainer were used to set up both unstimulated and stimulated whole‐blood cultures in Falcon 12 × 75 mm polystyrene tubes with caps (BD Biosciences, Auckland, New Zealand). Zero or 20 μl of the multi‐antigen working vaccine (1:100) was added to 200 μl of heparinised whole blood, giving a final stimulant concentration of 1:1000, before being incubated for 24 h at 37°C and 5% CO_2_ (Midi 40 CO_2_ incubator, Thermo Fisher Scientific, Waltham, MA, USA). The stimulant was a commercially available multi‐antigen vaccine containing diphtheria toxoid, tetanus toxoid, *Bordetella pertussis* antigens, hepatitis B surface antigen, poliovirus and *Haemophilus influenzae*, and was used to elicit a recall immune response. The 1:1000 stimulant dilution was chosen based on previous titration work/pilot work performed in our lab to illicit optimum CD69 expression on NK cells.

### 2.7 Natural Killer cell count and CD69 expression

After 24 h of incubation, peripheral blood cells were labelled with a cocktail of Pharmingen monoclonal antibodies (BD Biosciences) against human lymphocyte cell surface markers, as follows: 5 μl fluorescein isothiocyanate (FITC) conjugated anti‐CD4, 5 μl *R*‐phycoerythrin‐Cy5 (PE‐Cy5) conjugated anti‐CD8 and 10 μl PE conjugated anti‐CD69. The other mixture contained 5 μl FITC conjugated anti‐CD3, 20 μl PE‐Cy5 conjugated anti‐CD56 and 10 μl PE conjugated anti‐CD69. All monoclonal antibodies were titrated previously to obtain optimum fluorescence signalling with lowest antibody volume. All samples were then vortexed and placed on ice for 20 min, after which erythrocytes were lysed and leukocytes fixed using FACS Lyse (BD Biosciences). Leukocytes were then washed once in 3 ml ice cold phosphate‐buffered saline (PBS) containing 0.1% bovine serum albumin (BSA) and 2 mM EDTA (PBS–BSA–EDTA) before being resuspended in 400 μl PBS–BSA–EDTA. Samples were stored at 4°C for no more than 1 h before being run on a FACS Calibur flow cytometer (three‐color flow cytometric analysis) with Cell Quest analysis software (BD Biosciences).

**FIGURE 1:**
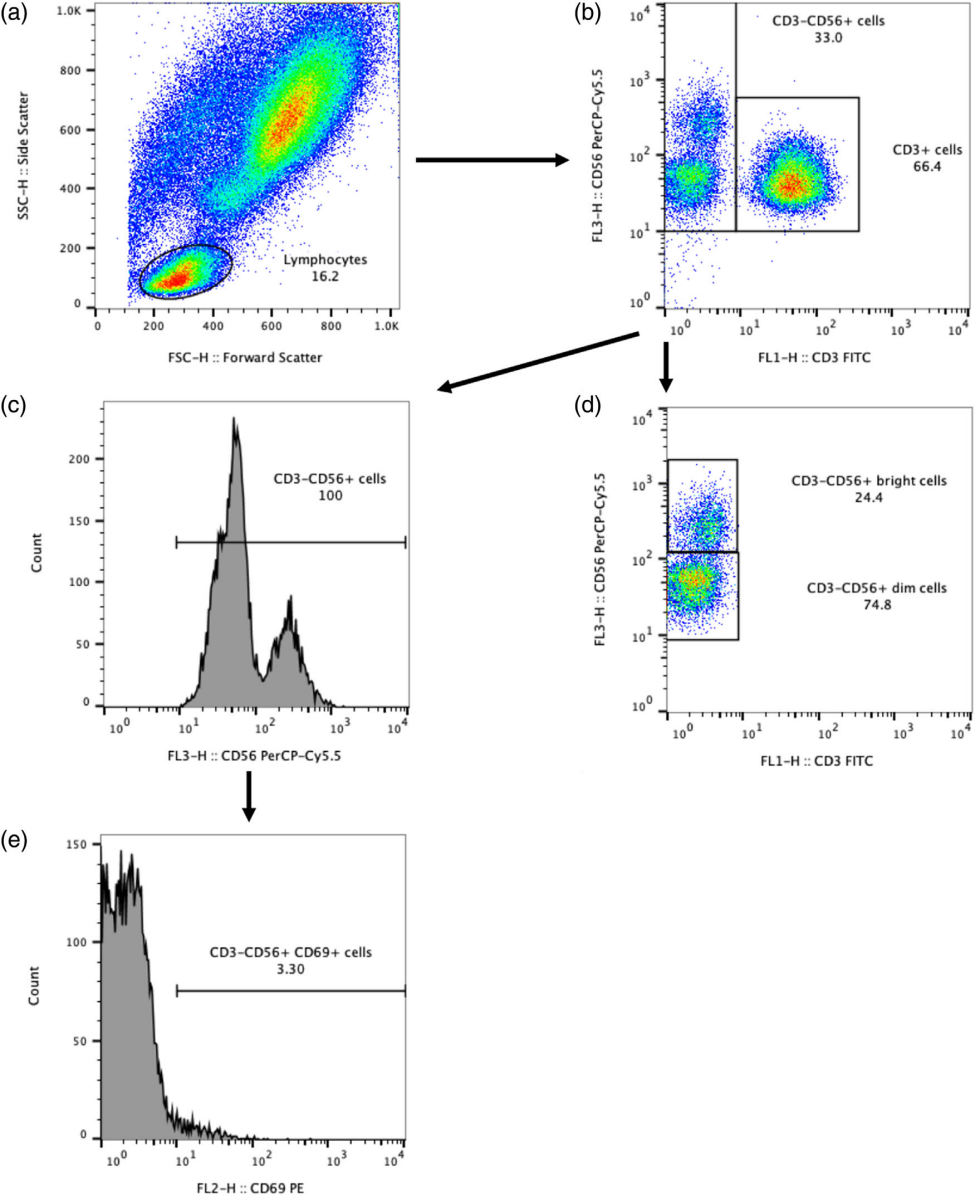
Gating strategy for natural killer (NK) cells’ subsets and CD3^+^CD56^+^CD69^+^ cells. (a) Peripheral blood events were measured against forward and side scatter parameters, and total lymphocytes were selected. (b) CD3^+^ cells were identified and removed from CD56^+^ analysis. (c) CD3^−^CD56^+^ bright and dim cells were subsequently identified. (d) Histogram plots of CD56 cells (negative for CD3) to identify all CD3^−^CD56^+^ cells. (e) Histogram plots of CD69 cells (negative for CD3 and positive for CD56) to identify CD3^−^CD56^+^CD69^+^ cells.

Standard gating procedures using side scatter versus forward scatter plots were used on a typical subject to gate the lymphocyte population. An unstained unstimulated sample was used to set quadrant boundaries to allow accurate acquisition of stained unstimulated samples. This procedure was repeated with unstained stimulated samples. All samples were set to collect 30,000 lymphocyte events per analysis. The flow cytometer was calibrated using caliBRITE beads (BD Biosciences) at the start of the study. CD3^+^ (T‐cell region) and CD56^+^ (NK cell region) populations were acquired on quadrant dot plots of FL1 (CD3^+^ FITC) and FL3 (CD56^+^ PE‐Cy5), along with quadrant dot plots of FL1 (CD3^+^) and FL2 (CD69^+^ PE) and quadrant dot plots of FL3 (CD56^+^) and FL2 (CD69^+^). These data were displayed as histogram plots and the percentage of CD3^−^ cells (total lymphocyte region minus the CD3^+^ region) expressing CD56^+^ was derived, and from this the percentage of total lymphocytes that were CD3^−^CD56^+^ was determined. CD3^−^CD56^+^ cells were gated into a separate region and CD69^+^ histogram plots of the cells were used to calculate the percentage expression and geometric mean fluorescence intensity (GMFI) of CD69 on CD3^−^CD56^+^ cells. CD69 expression on CD56^dim^ and CD56^bright^ cells were not determined as CD69 expression and CD56^bright^ cell counts were low on unstimulated samples. CD3^−^CD56^+^ cells were calculated by multiplying the percentage of cells with the absolute lymphocyte count, corrected for plasma volume changes. The number of CD3^−^CD56^+^ cells expressing CD69 was determined by multiplying the percentage of cells expressing CD69 by CD3^−^CD56^+^ cells. Figure 1 provides further detail of gating strategies for NK cells.

### 2.8 Data analysis

One participant was removed from all analyses as their T‐cell population could not be identified (thus erroneously conflating their NK cell percentage), giving a total sample size of *n* = 7. All statistical analyses and visualisations were performed in R version 3.6.3 with RStudio version 1.1.463. Data were analysed using linear mixed models with restricted maximum likelihood in the R package ‘lme4’. Fixed effect factors included diet (2 levels, KD or HD) and time point (3 levels, pre‐exercise, post‐exercise and 1‐h post‐exercise) and a random intercept for participant was included to adjust for inter‐individual homogeneity. Models were controlled for diet order (2 levels). Normality of distribution and homoscedasticity of the model's residuals were determined by visual inspection of Q–Q plots; if violated, data were either log, square‐root or inverse transformed and assessed for best fit prior to extracting *P*‐values. *P*‐values for fixed‐effects factors were obtained using a type II Wald F test with Kenward–Roger degrees of freedom in the R package ‘car’. *P*‐values for pairwise comparisons were obtained using Holm adjustment for multiplicity in the R package ‘emmeans’. Significance was inferred when *P* ≤ 0.05.

## 3 RESULTS

### 3.1 Circulating CD3^−^CD56^+^ natural killer cell count

**FIGURE 2:**
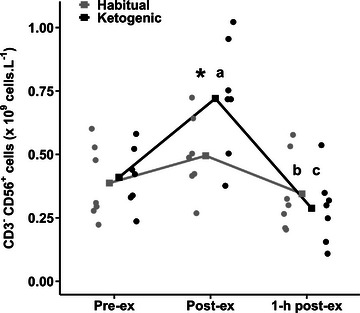
Circulating CD3^−^CD56^+^ cell counts in the habitual and ketogenic diet conditions. Values are presented as raw mean and individual responses for *n* = 7 participants. Diet × time point interaction: higher in the ketogenic diet (KD) compared with the habitual diet (HD) condition for time point (^*^ *P* < 0.05); higher at post‐exercise compared with pre‐exercise in the KD (^a^ *P* < 0.05); lower at 1 h post‐ compared with post‐ and pre‐exercise in the KD (^b^ *P* < 0.05); and lower at 1 h post‐exercise compared with post‐exercise in the HD (^c^ *P* < 0.05).

There was a diet × time point interaction for circulating CD3^−^CD56^+^ NK cell count (*P* = 0.004; Figure 2). Cell counts were similar between dietary conditions at pre‐exercise (*P* = 0.661). In the KD, cell counts increased from pre‐ to post‐exercise (*P* < 0.0001) and were 46% higher compared with the HD at post‐exercise (*P* = 0.0004). Cell counts declined by ∼60% in the KD from post‐exercise to 1‐h post‐exercise (*P* < 0.0001) to below pre‐exercise counts (*P* = 0.040). Cell counts also declined by ∼30% from post‐exercise to 1 h post‐exercise in the HD (*P* = 0.040) and remained similar to the KD at 1‐h post‐exercise (*P* = 0.350).

### 3.2 CD3^−^CD56^dim^ and CD3^−^CD56^bright^ cell subsets

**FIGURE 3:**
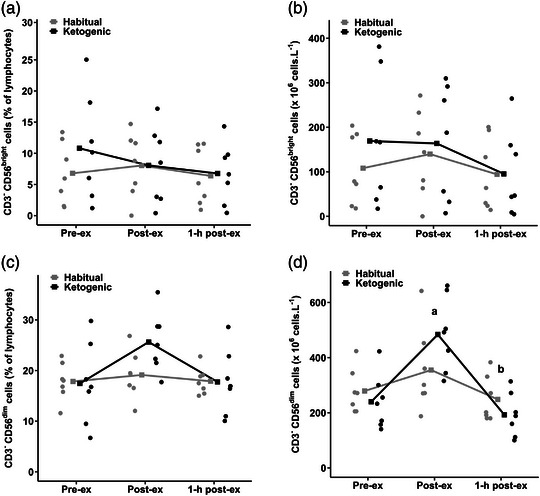
(a) CD56^bright^ subset percentage of total lymphocytes, (b) CD56^bright^ subset cell count, (c) CD56^dim^ subset percentage of total lymphocytes, and (d) CD56^dim^ subset cell count in the habitual and ketogenic diet conditions. Values are presented as raw mean and individual responses for *n* = 7 participants. Effect of time point: higher at post‐ compared with pre‐exercise (^a^ *P* < 0.05); and lower at 1‐h post‐ compared with post‐exercise (^b^ *P* < 0.05).

There were no diet × time point interactions (*P* = 0.420 and *P* = 0.542) or main effects of diet (*P* = 0.237 and *P* = 0.151) or time point (*P* = 0.402 and *P* = 0.104) for CD56^bright^ subset percentage of total lymphocytes and CD56^bright^ subset cell count, respectively (Figure 3a,b). There was a main effect of time point for CD56^dim^ subset percentage of total lymphocytes (*P* = 0.033; Figure 3c); however, post‐hoc analysis revealed no differences between time points (i.e., pre‐ vs. post‐exercise, *P* = 0.062; post‐ vs. 1 h post‐exercise, *P* = 0.062; and pre‐ vs. 1 h post‐exercise, *P* = 0.929). A diet × time point interaction for CD56^dim^ subset count was non‐significant (*P* = 0.054), but there was a main effect of time point for CD56^dim^ subset count (*P* < 0.0001; Figure 3d), which increased by ∼62% from pre‐ to post‐exercise (*P* = 0.001), then declined by ∼47% from post‐ to 1‐h post‐exercise (*P* = 0.0001).

### 3.3 CD3^−^CD56^+^ cells expressing CD69

**FIGURE 4:**
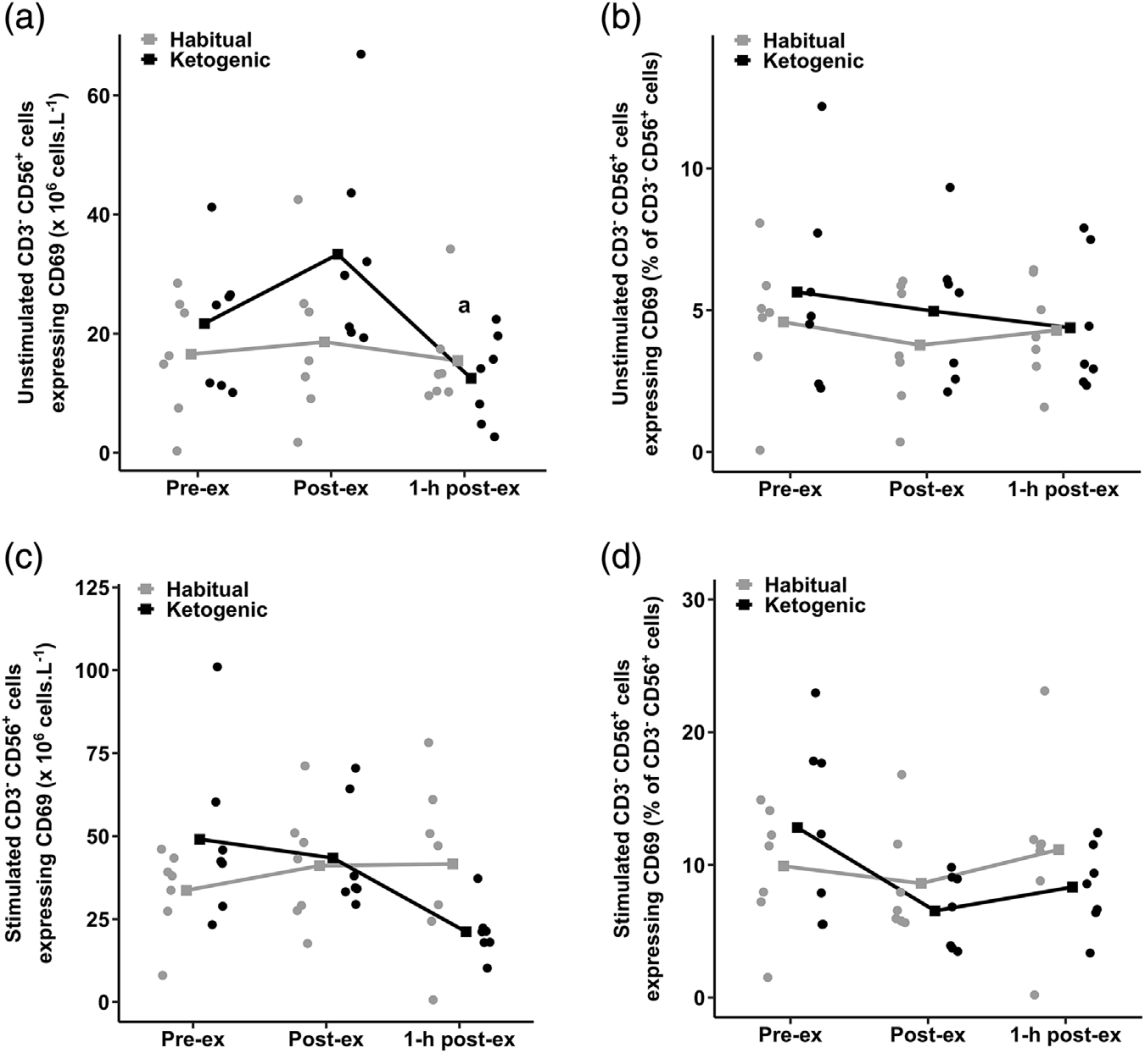
(a) Number of unstimulated CD3^−^CD56^+^ cells expressing CD69, (b) unstimulated CD3^−^CD56^+^ cells expressing CD69 as a percentage of total CD3^−^CD56^+^ cells, (c) number of antigen‐stimulated CD3^−^CD56^+^ cells expressing CD69, and (d) antigen‐stimulated CD3^−^CD56^+^ cells expressing CD69 as a percentage of total CD3^−^CD56^+^ cells in the habitual and ketogenic diet trials. Values are presented as raw mean and individual responses for *n* = 7 participants. Main effect of time point: lower at 1‐h post‐ compared with post‐exercise (^a^ *P* < 0.05).

There was an effect of time point for unstimulated circulating CD3^−^CD56^+^ cells expressing CD69 (*P* = 0.049; Figure 4a), which declined by ∼46% from post‐ to 1‐h post‐exercise (*P* = 0.045); however, there was no diet × time point interaction (*P* = 0.758) or main effects of diet (*P* = 0.240) or time point (*P* = 0.562) for unstimulated CD3^−^CD56^+^ expressing CD69 as a percentage of total CD3^−^CD56^+^ cells (Figure 4b). There was also no diet × time point interactions (*P* = 0.055 and *P* = 0.163) or main effects of diet (*P* = 0.999 and *P* = 0.676) or time point (*P* = 0.257 and *P* = 0.072) for antigen‐stimulated circulating CD3^−^CD56^+^ cells expressing CD69 (Figure 4c) and antigen‐stimulated CD3^−^CD56^+^ expressing CD69 as a percentage of total CD3^−^CD56^+^ cells (Figure 4d), respectively.

### 3.4 GMFI of CD69 expression on CD3^−^CD56^+^ cells

**FIGURE 5:**
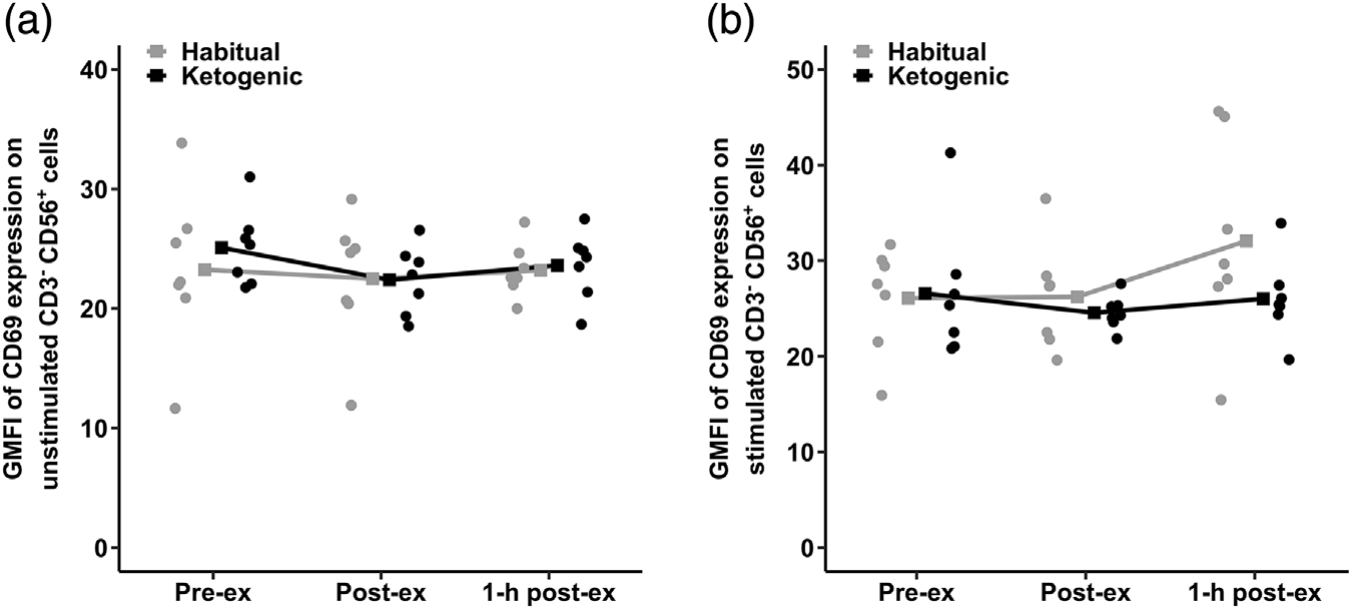
GMFI of CD69 expression on (a) unstimulated CD3^−^CD56^+^ cells and (b) antigen‐stimulated CD3^−^CD56^+^ cells in the habitual and ketogenic diet conditions. Values are presented as raw mean and individual responses for *n* = 7 participants.

There were no diet × time point interactions (*P* = 0.675 and *P* = 0.233) or main effects of diet (*P* = 0.504 and *P* = 0.216) or time point (*P* = 0.592 and *P* = 0.155) for the GMFI of CD69 expression on unstimulated and antigen‐stimulated CD3^−^CD56^+^ cells, respectively (Figure 5a,b).

## 4 DISCUSSION

We examined the effect of a 31‐day KD compared with a habitual CHO‐based diet on circulating CD3^−^CD56^+^ NK cells, the CD56^bright^ and CD56^dim^ subset counts, and in vitro unstimulated and antigen‐stimulated CD3^−^CD56^+^ NK cell activation in response to exhaustive moderate‐intensity running (∼3.5‐4 h, ∼45–50 km). Our primary observations were partly in support of our hypotheses and include: (1) the KD augmented the transient increase of circulating CD3^−^CD56^+^ cell counts immediately post‐exercise, which declined to below pre‐exercise counts at 1‐h post‐exercise; (2) the CD56^dim^ subset appeared the predominant subset shifting circulating NK cell counts; (3) circulating unstimulated CD3^−^CD56^+^ cells expressing CD69 were lower 1‐h post‐exercise compared with post‐exercise, but not when antigen‐stimulated; and (4) there was no change in the antigen‐stimulated CD69 expression (per cell expression of activation) in response to exercise or diet. These findings suggest that the KD amplifies the biphasic NK cell responses to prolonged exhaustive exercise, which appeared to be underpinned by the CD56^dim^ subset, but does not influence the ability of NK cells to activate to an antigenic challenge during recovery.

Adaptation to a KD shifts substrate utilisation toward fat to reduce the contribution of the body's finite stores of CHO to energy production during endurance exercise (Shaw et al., 2020). This is a practice that has grown in popularity over recent years within the athletic population, with varied performance outcomes being demonstrated. We further polarised substrate availability and metabolism in our study by implementing acute pre‐exercise and during‐exercise fuelling techniques. As reported in our previous study, blood ketone body and cortisol concentration increased in the KD condition, while blood glucose concentration remained similar between KD and HD conditions (Shaw et al., 2021), suggesting blood glucose concentration was unlikely to be a cause of immune differences between dietary conditions. Despite not measuring the catecholamine response, we have previously demonstrated that adaptation to a KD increased autonomic stress (suggestive of an increased catecholamine response) indirectly via reductions in resting heart rate variability (Maunder et al., 2021), which is consistent with other studies investigating KDs (Langfort et al., 1996, 1997). Considering these marked physiological differences between conditions, alterations in circulating NK cell number and function in response to exercise were expected following adaptation to the KD.

Exercise elicited a biphasic circulating NK cell count response, with an increase from pre‐ to post‐exercise, followed by a decline during the 1‐h recovery period. The trafficking of NK cells is a primary contributor to the classical exercise‐induced lymphocytosis and lymphopenia, which we observed in our previous study (Shaw et al., 2021). This was likely due to lymphocyte mobilisation from secondary lymphoid tissue, followed by redistribution to peripheral tissues (Gleeson & Bishop, 2005). A similar pattern occurred for the CD56^dim^ subset, whereas the CD56^bright^ subset was unaltered. This preferential mobilisation of the more cytotoxic CD56^dim^ subset is similar to other studies (Campbell et al., 2009; Timmons et al., 2006), whereas the CD56^bright^ subset, which have a predominant regulatory role, are relatively unaffected by exercise and typically recirculate between the blood and secondary lymphoid tissues (Cooper et al., 2001). Prolonged moderate‐intensity exercise, compared with short high‐intensity exercise, may also have less effect on the CD56^bright^ subset (Gannon et al., 1997); therefore, it is unsurprising that the circulating CD56^bright^ subset was unaltered in the present study. These findings highlight the need to report both circulating CD56^bright^ and CD56^dim^ subsets to better understand circulating NK cell function and mobilisation with exercise.

The KD amplified the NK cell count response to exercise. Despite the KD having no clear influence on the exercise‐induced changes of the CD56^dim^ subset (*P* = 0.054), this cytotoxic cell subset seemed likely to underpin this effect, particularly due to having a larger response to exercise. Similarly, short‐term low‐CHO diets (Mitchell et al., 1998) and exercise without CHO supplementation (Henson et al., 1999) also appear to exacerbate the biphasic NK cell response to exercise. In our study, the increase in CD56^dim^ cells also appears higher when exercising without CHO supplementation (Timmons et al., 2006), which is likely due to a greater catecholamine response preferentially mobilising and redistributing cytotoxic lymphocytes via stimulation of β_2_‐adrenergic receptors that are highly expressed on these cells (Dimitrov et al., 2010; Maisel et al., 1990). In contrast, post‐exercise reductions in the CD56^dim^ subset have been down to be exacerbated with CHO supplementation (Timmons et al., 2006). This was potentially due to selective margination of the CD56^dim^ subset, which could be due to increased leukocyte adhesion to endothelium through upregulation of adhesion proteins (Morigi et al., 1998), with CD56^dim^ cells more rapidly adherent than CD56^bright^ cells (Vujanovic et al., 1993). In our study, the lack of differences in blood glucose concentration between conditions may explain why we did not observe this.

Only exercise influenced the number of unstimulated circulating NK cells expressing CD69, which declined during 1 h of recovery. However, following antigen stimulation, this effect was abrogated, suggesting the NK cells retained their ability to activate. The greater autonomic stress response in the KD condition (Langfort et al., 1996) was expected to increase the mobilisation of NK cells primed for effector functions (i.e., CD69^+^) into circulation following exercise; however, there were no effects of diet on the number of NK cells expressing CD69 or the intensity of CD69 expression. It is possible that despite increased circulating cytotoxic cells, alternative mechanisms masked potential effects of diet and exercise. For example, in our previous study (Shaw et al., 2021), cortisol and antigen‐stimulated whole blood IL‐10 production were higher in the KD condition, which may have led to an immunosuppressive environment post‐exercise. Nevertheless, higher cortisol concentrations (following a high dose of caffeine) have been reported to not alter the number of antigen‐stimulated NK cells expressing CD69 in response to 90 min of strenuous exercise (Fletcher & Bishop, 2011).

The applicability and implications of our findings may be limited due to the characteristics of the sample population and methods employed. Endurance trained athletes that can maintain moderate‐intensity exercise for several hours are not representative of the general population, but were recruited for two primary reasons: (1) the nature of their training was more likely to supress immune function (Walsh et al., 2011); and (2) they are theoretically more metabolically endowed to gain an endurance benefit from adapting to a KD than their less trained counterparts. As such, it is difficult to ascertain if these same patterns of response would be seen amongst the general population undertaking less exhaustive exercise. Females were not included in the current study, and as such we are unable to translate these findings; however, previous research suggests exercise‐induced changes in circulating NK cell number and function can be consistent across sexes (Moyna, 1996). The short recovery period (i.e., 1‐h post‐exercise) may also not reflect the hypothesised ‘open‐window’ of immune suppression that can last several hours, and circulating NK cell count and function may not reflect what is occurring in peripheral tissues, where immune cells are more likely to encounter a pathogenic challenge. For example, cytotoxic NK cells typically mobilise and egress to a greater magnitude during exercise (Campbell et al., 2009), which can confer host protection. However, exercise‐induced trafficking patterns of (activated) NK cells remain unknown.

In conclusion, we demonstrated that adaptation to a 31‐day KD augments the biphasic circulating NK cell count response to exercise, which appeared to be due to a greater mobilisation and redistribution of the cytotoxic CD56^dim^ subset. However, the KD had no effect on circulating NK cells’ ability to activate (CD69^+^) in response to in vitro antigen stimulation. The exercise protocol also lowered the number of unstimulated NK cells expressing CD69 1‐h post‐exercise, although this effect was abolished following in vitro antigen stimulation. Overall, the KD appears to uniquely modulate circulating counts of NK cells following prolonged exhaustive exercise but not their ability to activate to antigen stimulation. This suggests the KD has little effect on the functional capacity of this aspect of the innate arm of the immune system and there is little impact of this practice on athletes’ susceptibility to viral infection via this immune measure.

## AUTHOR CONTRIBUTIONS

David M. Shaw and Deborah K. Dulson conceptualised the study; David M. Shaw implemented the study and collected all samples; Ed Maunder, Lauren Keaney and Deborah K. Dulson conducted the Flow Cytometry; David M. Shaw performed the statistical analyses; David M. Shaw wrote the first draft of the manuscript; David M. Shaw, Lauren Keaney, Ed Maunder and Deborah K. Dulson wrote the final version of the manuscript. All authors have read and approved the final version of this manuscript and agree to be accountable for all aspects of the work in ensuring that questions related to the accuracy or integrity of any part of the work are appropriately investigated and resolved. All persons designated as authors qualify for authorship, and all those who qualify for authorship are listed.

## CONFLICT OF INTEREST

None declared.

## FUNDING INFORMATION

The authors recieved no funding for this research.

## Supporting information



Statistical Summary Document

Raw Data

## Data Availability

Raw data are available as Supporting Information.
